# All‐Inorganic Manganese‐Based CsMnCl_3_ Nanocrystals for X‐Ray Imaging

**DOI:** 10.1002/advs.202201354

**Published:** 2022-04-24

**Authors:** Lin‐Quan Guan, Shuo Shi, Xiao‐Wei Niu, Shi‐Chen Guo, Jian Zhao, Tian‐Meng Ji, Hao Dong, Feng‐Yan Jia, Jia‐Wen Xiao, Ling‐Dong Sun, Chun‐Hua Yan

**Affiliations:** ^1^ Beijing National Laboratory for Molecular Sciences State Key Laboratory of Rare Earth Materials Chemistry and Applications PKU‐HKU Joint Laboratory in Rare Earth Materials and Bioinorganic Chemistry College of Chemistry and Molecular Engineering Peking University Beijing 100871 P. R. China; ^2^ Institute of Microstructure and Property of Advanced Materials Beijing Key Lab of Microstructure and Property of Advanced Materials Faculty of Materials and Manufacturing Beijing University of Technology Beijing 100124 P. R. China; ^3^ College of Chemistry and Chemical Engineering Lanzhou University Lanzhou 730000 P. R. China

**Keywords:** Mn‐based perovskites, nanocrystals, self‐absorption free, X‐ray imaging

## Abstract

Lead‐based halide perovskite nanomaterials with excellent optical properties have aroused great attention in the fields of solar cells, light‐emitting diodes, lasing, X‐ray imaging, etc. However, the toxicity of lead prompts researchers to develop alternatives to cut down the usage of lead. Herein, all‐inorganic manganese‐based perovskite derivatives, CsMnCl_3_ nanocrystals (NCs), with uniform size and morphology have been synthesized successfully via a modified hot‐injection method. These NCs have a direct bandgap of 4.08 eV and a broadband emission centered at 660 nm. Through introducing modicum lead (1%) into the CsMnCl_3_ NCs, the photoluminescence intensity greatly improves, and the quantum yield (PLQY) increases from 0.7% to 21%. Furthermore, the CsMnCl_3_:1%Pb NCs feature high‐efficiency of X‐ray absorption and radioluminescence, which make these NCs promising candidates for X‐ray imaging.

## Graphical abstract

All‐inorganic manganese‐based perovskite derivatives, CsMnCl_3_ nanocrystals, are synthesized and their optical properties are investigated. After introducing modicum lead into the CsMnCl_3_ NCs, the photoluminescence quantum yield increases up to 21%. These nanocrystals can convert X‐rays into visible lights. And a potential X‐ray imaging application is demonstrated.

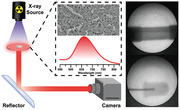

## 1 Introduction

Lead‐based halide perovskite materials, with superior optoelectronic properties such as large absorption coefficient, tunable bandgap, and high PLQY,^[^ ^1^ ^]^ have attracted tremendous attention in the last decade and undergo a great revolution in many fields, from solar energy conversion,^[^ ^2^, ^3^, ^4^ ^]^ optoelectronic devices^[^ ^5^, ^6^ ^]^ to lasing^[^ ^7^, ^8^ ^]^ and X‐ray scintillators.^[^ ^9^, ^10^, ^11^, ^12^ ^]^ Unfortunately, the overuse of toxic lead and poor stability against moisture, oxygen, high temperature, and electron beam, impede the practical applications of these halide perovskites.^[^ ^13^ ^]^

It is highly desired to replace toxic lead with other nontoxic or less‐toxic component while maintaining the excellent optical properties. The nontoxic tin, locating in the same group of the periodic table, has a similar electronic configuration and chemical properties to that of lead. However, tin‐based perovskite, CsSnX_3_ (X = Cl, Br, I) NCs, with a relatively low PLQY (0.14%), are extremely unstable under ambient conditions for the quick oxidation of Sn^2+^ to Sn^4+^.^[^ ^14^ ^]^ Recently, rare‐earth (RE) based perovskites CsEuCl_3_ NCs were synthesized, but regretfully, the PLQY is still not satisfied.^[^ ^15^ ^]^ Aliovalent substitution by Bi^3+^ ions with formulas of Cs_3_Bi_2_X_9_ and Cs_3_BiX_6_ were also reported.^[^ ^16^, ^17^ ^]^ Nevertheless, owing to the indirect bandgap nature, their optical performance is far from expected. It is the same for the double perovskites, Cs_2_AgBiCl_6_ NCs^[^ ^18^ ^]^ and Cs_2_AgSbCl_6_ NCs.^[^ ^19^ ^]^ Although Cs_2_AgInCl_6_ NCs exhibit a direct bandgap, the PLQY is only 1.6%,^[^ ^20^ ^]^ which is unable to meet the needs of photoelectronic applications.

Another alternative approach to reduce the content of lead is partially replacing Pb^2+^ ions by nontoxic and stable metal ions while keeping the perovskite structure. Mn^2+^ ions, with prominent optical and magnetic properties, were used as dopants in II–VI and III–V semiconductor quantum dots (QDs),^[^ ^21^, ^22^, ^23^ ^]^ to provide a new radiative recombination pathway. The emission wavelength of Mn^2+^ ions can be tuned from green^[^ ^24^ ^]^ to near‐infrared^[^ ^25^ ^]^ region by changing the local coordination structure and crystal field. Moreover, the spin‐forbidden *d*–*d* transition nature also results in a long lifetime and large Stokes shift with negligible self‐absorption.^[^ ^23^ ^]^ The attractive characters of both perovskites and Mn^2+^ ions encouraged researchers to shed light on the Mn‐doped perovskite NCs.^[^ ^26^, ^27^ ^]^ Introducing Mn^2+^ ions into the perovskite lattice not only cuts down lead content but also improves the stability of NCs.^[^ ^28^ ^]^ However, the doping concentration is still low due to the vastly different ionic radius between Pb^2+^ (133 pm, six‐coordinate) and Mn^2+^ ions (97 pm, six‐coordinate),^[^ ^26^ ^]^ which impedes the development of less lead‐containing halide perovskite. And CsMnCl_3_ appeared as impurity for heavily Mn content CsPbCl_3_ NCs.^[^ ^29^, ^30^, ^31^ ^]^

Inspired by these progress, to synthesize and figure out the optical properties of Mn‐based metal halide compounds instead of doping is desired. It is fascinating that circularly polarized luminescence of organic–inorganic hybrid [K(dibenzo‐18‐crown‐6)]_2_MnCl_4_ crystals with a high PLQY has been reported.^[^ ^24^ ^]^ And (C_38_H_34_P_2_)MnBr_4_ single crystal, with a PLQY as high as 95%, was discovered as an efficient X‐ray scintillators with a large light yield.^[^ ^32^ ^]^ All‐inorganic lead‐free perovskite Cs_4_MnBi_2_Cl_12_ crystals, with bright orange emission, show potentials for LED and X‐ray imaging.^[^ ^33^ ^]^ And CsMnCl_3_·2H_2_O crystals exhibit a solvatochromic photoluminescence with DMF/DMAC treatment, which leads to a changed PL from red to green.^[^ ^34^ ^]^ However, current studies about Mn‐based halide compounds are limited to single crystal or micro‐sized powders. The synthesis of pure phase all‐inorganic cesium manganese chloride NCs has less been explored and a solution‐phase strategy to get well‐defined colloidal NCs has yet been reported.

Herein, novel Mn‐based perovskite derivatives, CsMnCl_3_ NCs, were fabricated. These NCs are uniform in size and morphology, and a broadband emission centered at 660 nm were detected. To further improve their light absorption ability, modicum Pb^2+^ ions doped NCs were synthesized. Benefit from the parity allowed [PbCl_6_]^4−^ absorption and effective energy transfer from [PbCl_6_]^4−^ to Mn^2+^ ions, the PL intensity enhanced monotonously with the Pb content, and the highest PLQY up to 21% is obtained for CsMnCl_3_:1%Pb. Furthermore, these modicum Pb^2+^ ions containing CsMnCl_3_ NCs gave out visible emission under X‐ray excitation. And a potential X‐ray imaging was demonstrated. This work offers a promising way in largely reducing lead contents but maintaining comparable luminescence for the perovskites.

## 2 Result and Discussion

**Figure 1:**
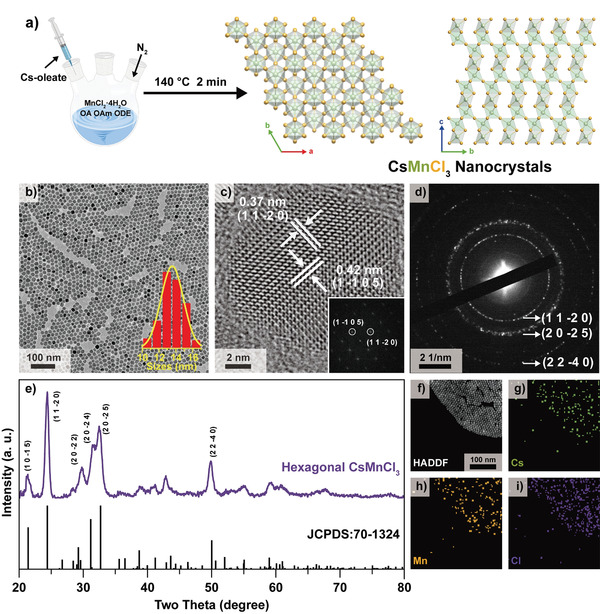
a) Schematic illustration of the synthesis and the structure of CsMnCl_3_ NCs. Characterization of CsMnCl_3_ NCs with b) TEM and c) HRTEM images, d) SAED and e) XRD patterns (with PDF card of hexagonal CsMnCl_3_, JCPDS No. 70–1324). Compositions of CsMnCl_3_ NCs from f) HAADF‐STEM image and g–i) EDS mapping of Cs, Mn, Cl elements. The inset of panels b) and c) are the corresponding size distribution and FFT pattern, respectively.

High‐quality CsMnCl_3_ NCs were synthesized via injecting Cs‐oleate into a mixed solution of oleic acid, oleylamine, and 1‐octadecene with excess MnCl_2_·4H_2_O at 140 °C, a modified hot‐injection solution approach.^[^ ^1^ ^]^ The volume ratio of oleic acid and oleylamine plays a key role in morphology and phase control, and the optimized one is 3:1 (**Figure** **1**a and details in the Supporting Information). In the structure of hexagonal CsMnCl_3_, [MnCl_6_]^4−^ octahedrons in CsMnCl_3_ are both corner and face sharing, which lead the skeleton of CsMnCl_3_ with quasi‐1D chain character (Figure 1a). Low magnified transmission electron microscopy (TEM) image reveals that the as‐synthesized NCs have spherical morphology with a narrow size distribution of 13.6 ± 1.3 nm (Figure 1b). The representative high‐resolution TEM (HRTEM) image and corresponding fast Fourier transform (FFT) pattern indicate these NCs are single crystalline with legible lattice distances of 0.37 and 0.42 nm, corresponding well to the (11‐20) and (1‐105) planes of the hexagonal CsMnCl_3_ (Figure 1c). Three distinct diffraction rings can be identified from the selected area electron diffraction (SAED) pattern, which match with the (11‐20), (20‐25), and (22‐40) planes of the hexagonal CsMnCl_3_ (Figure 1d). To further confirm the phase of these NCs, powder X‐ray diffraction (XRD) was carried out (Figure 1e), which can be well indexed into hexagonal CsMnCl_3_. As shown in Figure 1f–i, energy dispersive spectroscopy (EDS) mapping images manifest that the elements of Cs, Mn, and Cl distribute homogenously in these NCs, which can also be verified by the line scan profile (Figure S1, Supporting Information). The elemental contents of Cs, Mn, and Cl are 24.1%, 18.7%, and 57.2%, respectively, close to the stoichiometric ratio of CsMnCl_3_ (Figure S2, Supporting Information).

**Figure 2:**
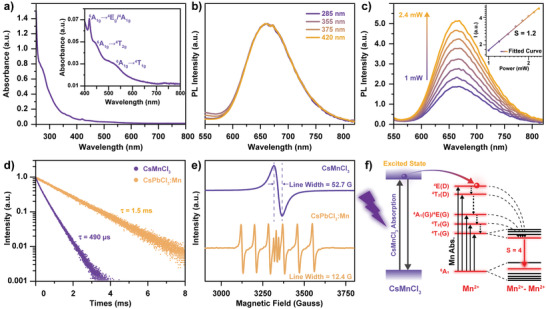
a) Absorption spectra of CsMnCl_3_ NCs and the corresponding b) PL spectra under excitation of Mn^2+^ ions (^4^E_d_, ^4^T_2d_, ^4^E_g_/^4^A_1g_,) at 355, 375, 420 nm, and CsMnCl_3_ at 285 nm. c) Power dependent PL spectra of CsMnCl_3_ NCs, the inset indicates a linear relationship between the excitation power and emission intensity. d) Time‐resolved PL spectra and e) EPR spectra taken at room temperature for CsMnCl_3_ and CsPbCl_3_:1.9%Mn. f) Schematic diagram of the PL mechanism of CsMnCl_3_ NCs.

The absorption spectrum of CsMnCl_3_ NCs was recorded as shown in **Figure** **2**a. A strong absorption beyond 300 nm as well as typical absorption of Mn^2+^ ions at 355, 375, 420, 450, and 540 nm from ^6^A_1g_ to ^4^E_d_, ^4^T_2d_, ^4^E_g_/^4^A_1g_, ^4^T_2g_, and ^4^T_1g_ (inset of Figure 2a), respectively, were discernible. The absorption of Mn^2+^ ions is feebler than that of the edge since the *d*–*d* transitions of Mn^2+^ ions is both spin and parity forbidden. Even if it is partially unblocked by breaking the centrosymmetry and the spin–orbit interaction, the absorption cross‐section coefficient is relatively small. The bandgap of CsMnCl_3_ NCs, 4.08 eV, can be obtained from the Tauc plot analysis according to the UV–vis absorption spectra (Figure S3, Supporting Information). To further clarify the optical properties of CsMnCl_3_ NCs, PL spectra were measured. As depicted in Figure 2b, these colloidal NCs give a broadband red emission at 660 nm, which is much redder than that of Mn‐doped CsPbCl_3_ NCs (600 nm, Figure S4, Supporting Information). No shift of PL peak position and change of full width at half‐maximum (FWHM) could be observed under the excitation of both CsMnCl_3_ at 285 nm and Mn^2+^ ions at 355, 375, and 420 nm (Figures S5 and S6, Supporting Information). Compared with the previous studies on the CsMnCl_3_ microcrystals,^[^ ^34^, ^35^ ^]^ the red emission of Mn^2+^ ions originated from the six‐coordinated configuration in [MnCl_6_]^4−^ octahedrons. In addition, the power‐dependent PL spectra (Figure 2c) show that the luminescence intensity is linear increased with the exciting power (inset of Figure 2c). This is well correlated with the luminescence of CsMnCl_3_⋅2H_2_O single crystal.^[^ ^34^ ^]^ The PL decay (Figure 2d) could be well‐fitted with a monoexponential function, and a lifetime of 490 µs was deduced, which is shorter than that of CsPbCl_3_:1.9%Mn NCs (1.5 ms). Furthermore, the luminescence intensity decreased ≈2% in 12 h under continuous irradiation at 285 nm (Figure S7, Supporting Information), showed a much improved photostability compared with that of halide perovskites.

Since in the structure of CsMnCl_3_ NCs, the [MnCl_6_]^4−^ clustered with face‐sharing and further linked by the corner Cl^−^, a significant magnetic coupling is expected for the nearest‐neighboring Mn^2+^ ions with a distance of 3.2 Å.^[^ ^25^, ^35^ ^]^ Electron paramagnetic resonance (EPR) spectra were recorded as shown in Figure 2e,a broad EPR signal, with a linewidth of 52.7 G is significant. This indicates a close interaction among Mn^2+^ ions in CsMnCl_3_ NCs. On the contrary, far‐isolated Mn^2+^ ions in the CsPbCl_3_:1.9% Mn NCs show six hyperfine narrow splitting with a linewidth of 12.4 G (Figure 2e). Typical temperature‐dependent PL spectra (Figure S8, Supporting Information) were measured and summarized in Figure S9 in the Supporting Information. The peak positions are not monotonous shift with temperature and a spinodal appeared at around 120 K. From the literatures, such a tendency in Mn‐doped NCs has been attributed to the Mn–Mn magnetic coupling.^[^ ^23^, ^36^ ^]^ Furthermore, the coupling of adjacent Mn^2+^ ions leads further splitting of ^6^A_1_ into six multiples (*S* = 0–5) and ^4^T_1_ into four (*S* = 1–4). The narrowed the energy gap between ^6^A_1_ and ^4^T_1_ (Figure S10, Supporting Information) induces the red shifted emissions from Mn^2+^ ions and accelerates the recombination rate.^[^ ^23^ ^]^ These are consistent with the PL spectra and lifetime variant tendency in Figure 2b,d and Figure S4 (Supporting Information). Based on the above investigations, a proposed PL mechanism is illuminated in Figure 2f for CsMnCl_3_ NCs.

**Figure 3:**
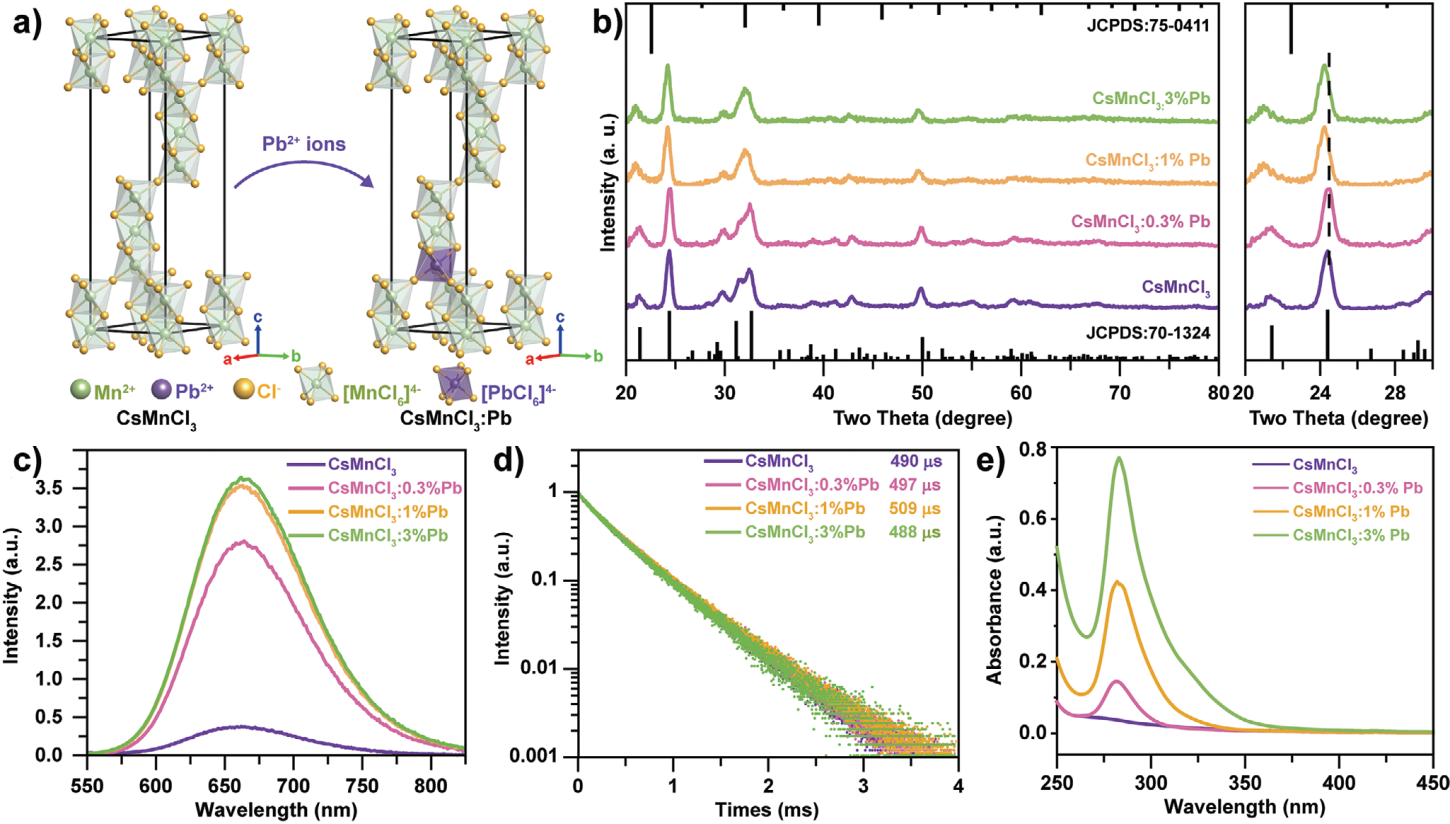
a) Schematic diagram of Pb^2+^ ions introduced into CsMnCl_3_ NCs. b) XRD patterns of the as‐prepared CsMnCl_3_:Pb NCs (hexagonal CsMnCl_3_, JCPDS No. 70–1324, and cubic CsPbCl_3_, JCPDS No. 75–0411). c) PL spectra, d) time‐resolved PL spectra, and e) absorption spectra of CsMnCl_3_ NCs with Pb^2+^ ions dopant of 0%, 0.3%, 1%, and 3%.

Composition regulation is crucial to optimize the properties of perovskite NCs.^[^ ^37^ ^]^ Since the 6s^2^ electronic configuration plays a key role in improving the optical performance of perovskites, a modicum amount of Pb^2+^ ions, a content of 0.3% to 3%, were tried to introduce into CsMnCl_3_ NCs (**Figure** **3**a). TEM images show all these samples are uniform sphere with a slight size variation from 13.6 to 15.7 nm (Figure S11, Supporting Information). The XRD patterns of all these CsMnCl_3_:Pb NCs manifest that the hexagonal structure is maintained and no impurity of cubic phase CsPbCl_3_ appears (Figure 3b). Meanwhile, due to the larger Pb^2+^ ion radius (133 pm, six‐coordinate) than that of Mn^2+^ (97 pm, six‐coordinate),^[^ ^26^ ^]^ the diffraction patterns shift to smaller angle, and the diffraction from (11‐20) shifts from 24.4° to 24.2° with 3% Pb doping (Figure 3b, right). This confirmed that Pb^2+^ ions are successful introduced into CsMnCl_3_ lattice without altering the structure, just resulting a small lattice expansion. And the Pb^2+^ ions concentration is close to the feeding one (Table S1, Supporting Information) from the results of inductively coupled plasma atomic emission spectroscopy (ICP‐AES). This may stem from the smaller binding energy of Pb–Cl (301 kJ mol^−1^) than that of Mn–Cl (338 kJ mol^−1^),^[^ ^26^ ^]^ favoring Pb^2+^ ions incorporation.

Similar to the undoped CsMnCl_3_ NCs, the Pb‐doped counterparts also give out a broadband emission at 660 nm. The PL intensity increases monotonously and about an order of magnitude enhancement is achieved as the Pb doping ratio reaches up to 3% (Figure 3c). Moreover, the highest PLQY is 21.1%, much higher than the undoped CsMnCl_3_ (0.7%, Table S2, Supporting Information). The time‐resolved PL spectra indicate all these samples have similar lifetimes with a small variation between 488 and 508 µs (Figure 3d), which is similar to that of CsMnCl_3_ NCs. To gain further insights into the enhanced emission for CsMnCl_3_:Pb NCs, the absorption spectra of these NCs was recorded in Figure 3e. The undoped CsMnCl_3_ NCs exhibit a weak exciton absorption at 285 nm. With the incorporation of Pb^2+^ ions, the absorption around 285 nm was greatly enhanced due to the parity allowed transition ^1^S_0_ → ^3^P_1_ of [PbCl_6_]^4−^ octahedrons. The enhanced absorption and effective energy transfer from [PbCl_6_]^4−^ to Mn^2+^ ions^[^ ^38^ ^]^ finally result in an increased PL intensity. The large Stokes shift between absorption and emission benefits applications in solid state for a negligible self‐absorption. In addition, the photostability is also evaluated and the photoluminescence intensity does not have obvious variation by continuous irradiation of these NCs in 12 h (Figure S12, Supporting Information). The strategy to improve the absorption probability is further verified for Bi‐doped CsMnCl_3_ NCs. On account of the transition ^1^S_0_ → ^3^P_1_ of [BiCl_6_]^3−^ octahedrons is parity allowed, the absorption around 330 nm of Bi‐doped CsMnCl_3_ NCs (Figure S13, Supporting Information) was gradually enhanced with an increased Bi^3+^ ions concentration (Figure S14, Supporting Information). With energy transfer from [BiCl_6_]^3−^ to Mn^2+^ ions, the CsMnCl_3_:Bi NCs also exhibit an enhanced luminescence (Figure S15, Supporting Information).

**Figure 4:**
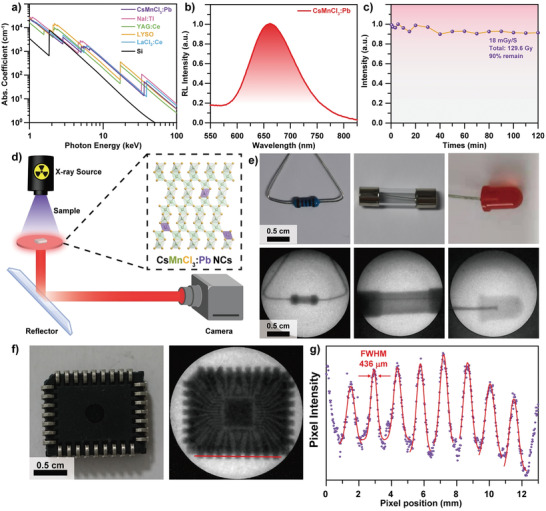
a) Absorption coefficients of CsMnCl_3_:Pb NCs and commercial scintillators as a function of X‐ray energy. b) Radioluminescence spectra of CsMnCl_3_:Pb NCs under the irradiation at 70 kV, 170 µA with average X‐Ray photon energy about 15 keV. c) The change of the radioluminescence intensity under continuous X‐ray excitation with a dose rate of 18 mGy s^−1^. d) Schematic illustration of X‐ray imaging configuration. e) Digital photographs of the target objects (resistance wire, fuse‐link, and light‐emitting diode) under natural light (upper row) and X‐ray irradiation (lower row). f) Digital photographs of a circuit board under natural light (left) and X‐ray irradiation (right). g) Spatial resolution via the point spread profile fitting.

Scintillators are widely used in detecting ionizing radiation, computed tomography and particle physics.^[^ ^10^, ^39^ ^]^ Compared with the conventional commercial scintillators, colloidal NCs synthesized at a relatively low temperature with solution processability are attractive for a high‐contrast X‐ray in vivo bioimaging^[^ ^40^ ^]^ and flexible X‐ray detector.^[^ ^41^ ^]^ Recently, perovskite NCs with advantages in large atomic number and tunable emission color have been investigated for luminous scintillators.^[^ ^9^, ^10^, ^11^, ^12^ ^]^ However, the application are indefinite for the toxicity concern of lead.^[^ ^32^, ^33^ ^]^ Considering the high PLQY, good PL stability, negligible self‐absorption and less lead content, the CsMnCl_3_:1%Pb NCs were tested for potential X‐ray luminous detectors. It should be noticed that we ultimately choose 1% rather than 3% Pb doped NCs for they have a lower lead concentration and a comparable PL emission. We firstly investigated the X‐ray absorption ability^[^ ^42^ ^]^ of CsMnCl_3_:1%Pb NCs at the photon energy from 1 to 100 keV. The absorption of CsMnCl_3_:1%Pb NCs is comparable with the commercial scintillators, such as NaI:Tl, YAG:Ce, LYSO, LaCl_3_:Ce, and Si (**Figure** **4**a). The excellent X‐ray absorption ability could be attributed to the large atomic number of Cs^+^ and Pb^2+^ ions and relative high density (3.47 g cm^–3^) for the X‐ray absorption coefficient is proportional to the density and fourth power of effective atomic number. The X‐ray activated performance of CsMnCl_3_:Pb NCs were further examined by irradiation with an X‐ray tube (70 kV, 170 µA, *E*_strongest_ ≈ 8.45 keV and *E*_average_ ≈ 15 keV). The radioluminescence spectra are almost identical with the PL spectra, indicating the same radiative recombination pathway with that of UV irradiation (Figures 2b and 4b). And the broad emission band of CsMnCl_3_:1%Pb NCs centered at 660 nm is convenient to match the detector to improve the response efficiency. The red emission is nonoverlapped with conventional scintillating materials, which provides the possibility to couple them together towards heterostructured detectors.^[^ ^39^ ^]^

The light output is a parameter to evaluate scintillation property. Considering the large difference in size for nanoparticles and single crystals, the light output can be affected by the light scattering.^[^ ^43^ ^]^ With CsPbBr_3_ QDs as a reference, which have a similar size with CsMnCl_3_:Pb NCs and a known light yield about 21 000 ph MeV^−1^,^[^ ^10^, ^32^, ^44^ ^]^ the light yield of CsMnCl_3_:Pb NCs is estimated about 2500 ph MeV^−1^ (Figure S16, Supporting Information). The irradiation stability of CsMnCl_3_:1%Pb NCs was also investigated. With an X‐ray exposure of high dose rate (18 mGy s^−1^) under ambient condition for 2 h (total dose of 129.6 Gy), 90% of the initial level was remained (Figure 4c; Figure S17, Supporting Information), better than CsI:Tl (50% remained at 30 Gy)^[^ ^45^ ^]^ and lead‐free double perovskite scintillator, Cs_2_Ag_0.6_Na_0.4_InCl_6_:Bi (50% remained at 53 Gy).^[^ ^46^ ^]^

The ability of converting the X‐rays into visible lights motivates the X‐ray imaging application of CsMnCl_3_:1%Pb NCs. High quality image with no lag and high repetition rate is important for practical application.^[^ ^32^ ^]^ As indicated in Figure S18 (Supporting Information), the intensity decreased to the background level within 10 ms after ceasing the excitation for CsMnCl_3_:Pb NCs. This is in keeping with lifetime measurement (Figure 3d) and they are suitable for high contrast imaging. As a proof‐of‐concept, a purpose‐built system comprising an X‐ray tube and a commercial digital camera was set for X‐ray imaging studies (Figure 4d). The thickness of the nanocrystal pallet is about 240 µm, and most of the X‐ray photons could be absorbed (Figure S19, Supporting Information). As shown in Figure 4e,f, resistance wire, fuse‐link, light‐emitting diode, and circuit board were used to illuminate the X‐ray imaging ability. The outline of resistance wire and the threadlet in fuse‐link could be recognized clearly under X‐ray irradiation. The inner structures of the light‐emitting diode and circuit board could not be distinguished under natural light. Due to the difference X‐ray transmittance between metal and plastic, the legible inner configurations could be disclosed under X‐rays. The spatial resolution of the CsMnCl_3_:1%Pb scintillator could reach 4.3 line pairs per millimeter as shown in Figure S20 (Supporting Information), and this is comparable with lead‐free double perovskite scintillator, Cs_2_Ag_0.6_Na_0.4_In_0.85_Bi_0.15_Cl_6_ (4.4),^[^ ^47^ ^]^ and the commercial *a*‐Se based direct flat panel detector (4.75),^[^ ^48^ ^]^ but less than that of GOS (6.2)^[^ ^49^ ^]^ and CsPbBr_3_ QDs (9.6).^[^ ^49^ ^]^ It is anticipated that a better spatial resolution may acquire with a closer detection mode. And we also measured spatial resolution by fitting the point spread profile with Gaussian function.^[^ ^10^, ^32^ ^]^ The spatial resolution was determined as 436 µm (Figure 4g), which is comparable with another Mn‐based scintillator (C_38_H_34_P_2_)MnBr_4_.^[^ ^32^ ^]^ These primary results support the assumption and prospect that CsMnCl_3_:1%Pb NCs are good for X‐ray imaging.

## 3 Conclusion

In summary, we successfully synthesized novel Mn‐based perovskite derivatives, CsMnCl_3_ NCs, with narrow size distribution and uniform spherical morphology. Optical studies reveal that CsMnCl_3_ NCs have a direct bandgap of 4.08 eV and a broadband emission around 660 nm. After introducing modicum Pb^2+^ ions into the CsMnCl_3_, the PL intensity enhances and the PLQY can be further increased to 21%. Moreover, on account of high PLQY and negligible self‐absorption, CsMnCl_3_:Pb NCs present excellent X‐ray scintillation performance, which enables X‐ray imaging. This work provides a new class of low toxic perovskite derivative NCs while maintaining the excellent optical properties, and presents an opportunity toward new type of photoelectric materials.

## 4 Experimental Section

The details of experimental methods can be found in the Supporting Information

## Conflict of Interest

The authors declare no conflict of interest.

## Supporting information

Supporting Information

## Data Availability

The data that support the findings of this study are available from the corresponding author upon reasonable request.
