# Supplementary material for: All‐Inorganic Manganese‐Based CsMnCl3 Nanocrystals for X‐Ray Imaging
Source: Adv Sci (Weinh). 2022 Apr 24;9(18):2201354. doi: 10.1002/advs.202201354 (PMC9218781; doi:10.1002/advs.202201354)
Supplement: Supplementary file 1 — Supporting Information [file ADVS-9-2201354-s001.pdf]

ADVANCED SCIENCE
Open Access

Supporting Information

for *Adv. Sci.*, DOI 10.1002/advs.202201354

All-Inorganic Manganese-Based $CsMnCl_3$ Nanocrystals for X-Ray Imaging

Lin-Quan Guan, Shuo Shi, Xiao-Wei Niu, Shi-Chen Guo, Jian Zhao, Tian-Meng Ji, Hao Dong, Feng-Yan Jia, Jia-Wen Xiao, Ling-Dong Sun* and Chun-Hua Yan**

Supporting Information

All-Inorganic Manganese-Based $CsMnCl_3$ Nanocrystals for X-Ray Imaging

Lin-Quan Guan, Shuo Shi, Xiao-Wei Niu, Shi-Chen Guo, Jian Zhao, Tian-Meng Ji, Hao Dong, Feng-Yan Jia, Jia-Wen Xiao, Ling-Dong Sun,* and Chun-Hua Yan**

L.-Q. Guan, Dr. S. Shi, S.-C. Guo, J. Zhao, T.-M. Ji, Dr. H. Dong, F.-Y. Jia, Prof. L.-D. Sun, Prof. C.-H. Yan
Beijing National Laboratory for Molecular Sciences
State Key Laboratory of Rare Earth Materials Chemistry and Applications
PKU-HKU Joint Laboratory in Rare Earth Materials and Bioinorganic Chemistry
College of Chemistry and Molecular Engineering
Peking University
Beijing 100871, China
E-mail: yan@pku.edu.cn; sun@pku.edu.cn

X.-W. Niu, Prof. J.-W. Xiao
Institute of Microstructure and Properties of Advanced Materials
Beijing Key Lab of Microstructure and Property of Advanced Material
Faculty of Materials and Manufacturing
Beijing University of Technology
Beijing 100124, China
E-mail: xiaojw@bjut.edu.cn

Prof. C.-H. Yan
College of Chemistry and Chemical Engineering
Lanzhou University
Lanzhou 730000, China

Experimental Methods

Materials and Chemicals

Cesium carbonate (Cs_2CO_3, ≥99.9%) and Manganese chloride tetrahydrate ($MnCl_2·4H_2O$, A.R.) were purchased from Energy Chemical and Sinopharm Chemical Reagent Co., Ltd. Manganese acetate tetrahydrate ($Mn(CH_3COO)_2·4H_2O$, A.R.) and lead acetate trihydrate ($Pb(CH_3COO)_2·3H_2O$, A.R.) were purchased from Xilong Scientific Co., Ltd. Bismuth acetate ($Bi(CH_3COO)_3$, ≥99.99%) and polymethyl methacrylate (PMMA, MW ~35000) were procured from J&K and Sinopharm Chemical Reagent Co., Ltd., respectively. Lead chloride ($PbCl_2$, A.R.), lead bromide ($PbBr_2$, A.R.) were purchased from Aladdin Reagent Co., Ltd. Oleic acid (OA, >90%), oleylamine (OAm, 98%), and 1-octadecene (ODE, >90%) were obtained from Sigma-Aldrich, Acros, and J&K, respectively. Cyclohexane (A.R.), and ethyl acetate (CCER) are bought from Beijing Chemical Works. All chemicals were used as received without further purification.

Instrumentation

TEM and HRTEM images, SAED and HAADF-STEM images, EDS spectra, EDS line scan profile and EDS mapping images were taken with a JEOL JEM-2100F TEM operated at 200 kV. Powder X-ray Diffraction (XRD) patterns were recorded on a Bruker D2 PHASER diffractometer (Bruker, Germany), using Cu Kα radiation (λ = 1.5406 Å). Inductively coupled plasma atomic emission spectroscopy (ICP-AES) analysis was carried out with a Leeman Profile SPEC (Leeman, American). The UV-vis absorption spectra were measured on the UV-3600 Plus UV-vis-NIR spectrophotometer (Shimadzu, Japan). The steady-state PL spectra, time-resolved PL spectra were recorded on the FLS980 spectrometer (Edinburgh Instrument, England) equipped with continuous and microseconds xenon lamps (μF920H Xe flash lamp, 50 Hz). Temperature-dependent PL spectra (77−280 K) were performed by setting the sample in a cryostat (Optistat DN2, Oxford Instruments, England). Power-dependent PL spectra were

carried out using a Taiko picosecond diode laser (PicoQuant, Germany) as the excitation source with the excitation at 375 nm. The absolute quantum yield measurements were also recorded on the FLS980 spectrometer with an integrating sphere. Electron paramagnetic resonance (EPR) spectra were obtained on a Bruker Elexsys E580 X-band (9.4 GHz) EPR spectrometer (Bruker, Germany) at room temperature. Radioluminescence spectra were collected with a photomultiplier tube irradiated with a Moxtek X-ray tube (70 kV, 170 μA). The afterglow intensity was recorded by continuously irradiating 20 s under xenon lamp and then the afterglow signal was collected by FLS980 spectrometer with a 10 ms time interval.

Preparation of Cesium−Oleate Solution

The cesium-oleate solution was prepared according to the approach reported with slight modifications [1]. In brief, 0.207 g of Cs_2CO_3, 0.56 g of OA, and 7.9 g of ODE were loaded into a 50 mL three-neck flask and degassed under vacuum at 120 °C for 30 minutes and then heated up to 150 °C under N_2 atmosphere till a clear solution formed. The solution was then kept at 120 °C in N_2 atmosphere before use.

Preparation of $CsMnCl_3$ Nanocrystals

0.0744 g (0.376 mmol) $MnCl_2·4H_2O$, 1.335 g (1.5 mL) OA, 0.405 g (0.5 mL) OAm, and 7.9 g (10 mL) ODE were loaded into a 50 mL three-neck flask and degassed under vacuum at 120 °C for 5 minutes to remove the oxygen and moisture residue, together with the $MnCl_2·4H_2O$ precursors dissolved completely. Subsequently, the solution was heated to 140 ˚C under N_2 atmosphere. 0.8 mL hot Cs-oleate precursor solution was quickly injected into the above mixtures. The reaction was kept at 140 °C for 2 minutes and cooled down to room temperature with an ice bath. $CsMnCl_3$ nanocrystals can be collected by centrifugation at 8000 r.p.m. for 10 minutes and stored in 5 mL cyclohexane or wash with ethyl acetate to obtain solid powders for further use.

Preparation of Lead−Oleate and Bismuth−Oleate Solution

0.1426 g (0.376 mmol) $Pb(CH_3COO)_2·3H_2O$, 0.89 g (1 mL) OA, 0.81 g (1 mL) OAm, and 7.9 g (10 mL) ODE were mixed in a 50 mL three-neck flask and degassed under vacuum at 120 °C for 30 minutes until a transparent solution is formed. It was then cooled down to room temperature naturally, and lead-oleate solution was prepared.

0.1452 g (0.376 mmol) $Bi(CH_3COO)_3$, 1.51 g (1.7 mL) OA, 0.3 g (0.37 mL) OAm, and 4.74 g (6 mL) ODE were mixed in a 50 mL three-neck flask and degassed under vacuum at 150 °C for 30 minutes to form a clear bismuth-oleate solution.

Preparation of $CsMnCl_3$:x%Pb Nanocrystals

The synthesis of $CsMnCl_3$:x%Pb nanocrystals was similar to that of $CsMnCl_3$ nanocrystals. Except for 36 μL, 120 μL, and 360 μL Pb-oleate precursor solution was added into the mixtures, where the x of the three samples are 0.3, 1, and 3, respectively.

Preparation of $CsMnCl_3$:x%Bi Nanocrystals

The synthesis of $CsMnCl_3$:x%Bi nanocrystals was similar to that of $CsMnCl_3$ nanocrystals, except for 16 μL, 40 μL, and 80 μL Bi-oleate precursor solution was added into the mixtures to keep x as 0.2, 0.5, and 1, respectively.

Preparation of $CsPbCl_3$:Mn Nanocrystals

The synthesis of $CsPbCl_3$:Mn nanocrystals was according to the previous report[2]. In a typical synthesis, 0.051 g (0.188 mmol) $PbCl_2$, 0.0074 g (0.0376 mmol) $MnCl_2·4H_2O$, 0.445 g (0.5 mL) OA, 0.405 g (0.5 mL) OAm, and 3.95 g (5 mL) ODE were loaded into a 50 mL three-neck flask and degassed under vacuum at 120 °C for 30 minutes to remove the oxygen and moisture residue. Subsequently, the solution was heated to 140 ˚C under N_2 atmosphere. After the precursors dissolved completely, 0.4 mL hot Cs-oleate precursor solution was quickly injected into the above mixture to react at 140 °C for 10 seconds. It was then cooled down quickly with an ice bath. $CsPbCl_3$:Mn nanocrystals can be collected by centrifugation at 12000 r.p.m. for 10 minutes and stored in 5 mL cyclohexane.

Preparation of $CsPbBr_3$ Nanocrystals

The synthesis of $CsPbBr_3$ nanocrystals was according to the previous report[2]. In a typical synthesis, 0.067 g (0.188 mmol) $PbBr_2$, 0.89 g (1 mL) OA, 0.81 g (1 mL) OAm, and 3.95 g (5 mL) ODE were loaded into a 50 mL three-neck flask and degassed under vacuum at 120 °C for 30 minutes to remove the oxygen and moisture residue. Subsequently, the solution was heated to 160 ˚C under N_2 atmosphere. After the precursors dissolved completely, 0.4 mL hot Cs-oleate precursor solution was quickly injected into the above mixture to react at 160 °C for 10 seconds. It was then cooled down quickly with an ice bath. $CsPbBr_3$ nanocrystals can be collected by centrifugation at 12000 r.p.m. for 10 minutes and wash with ethyl acetate to obtain solid powders.

Fabrication of $CsMnCl_3$:Pb nanocrystals pallet

80 mg $CsMnCl_3$:Pb powders sample mixed with 20 mg PMMA throughly with grinding. The mixtures was then pressed into thin film using a hydraulic press at 20 T pressure for 5 minutes and the thickness is about 240 μm.

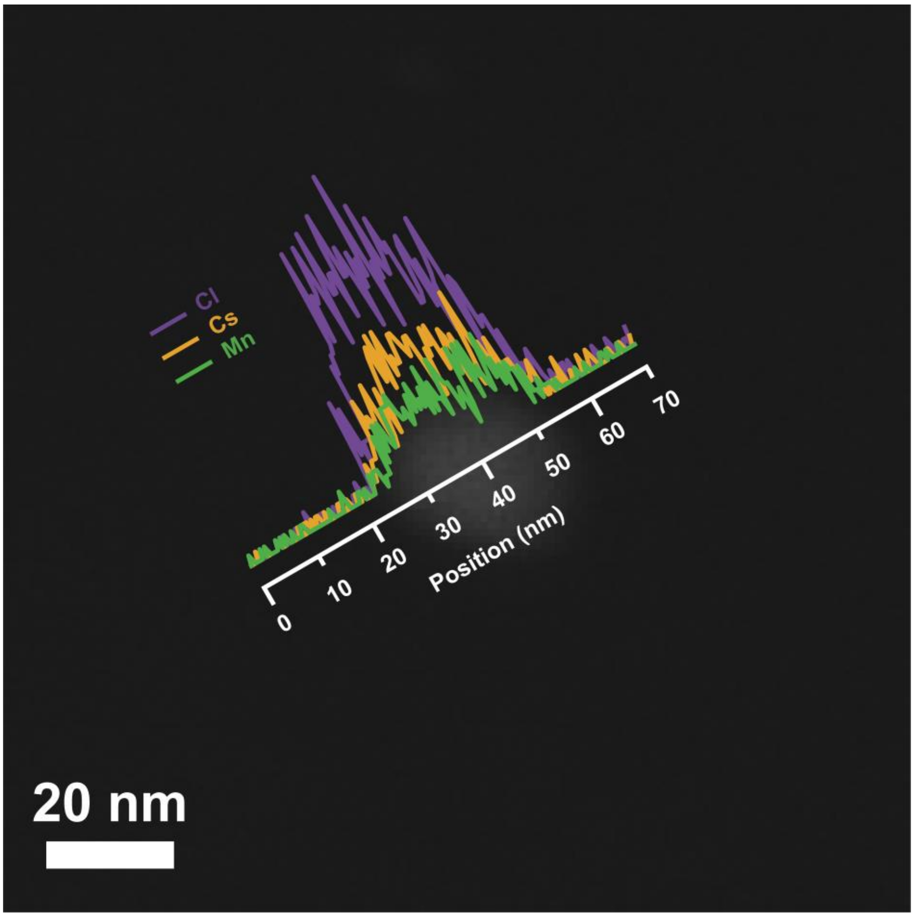

Figure S1. EDS line scan profiles of the $CsMnCl_3$ NCs.

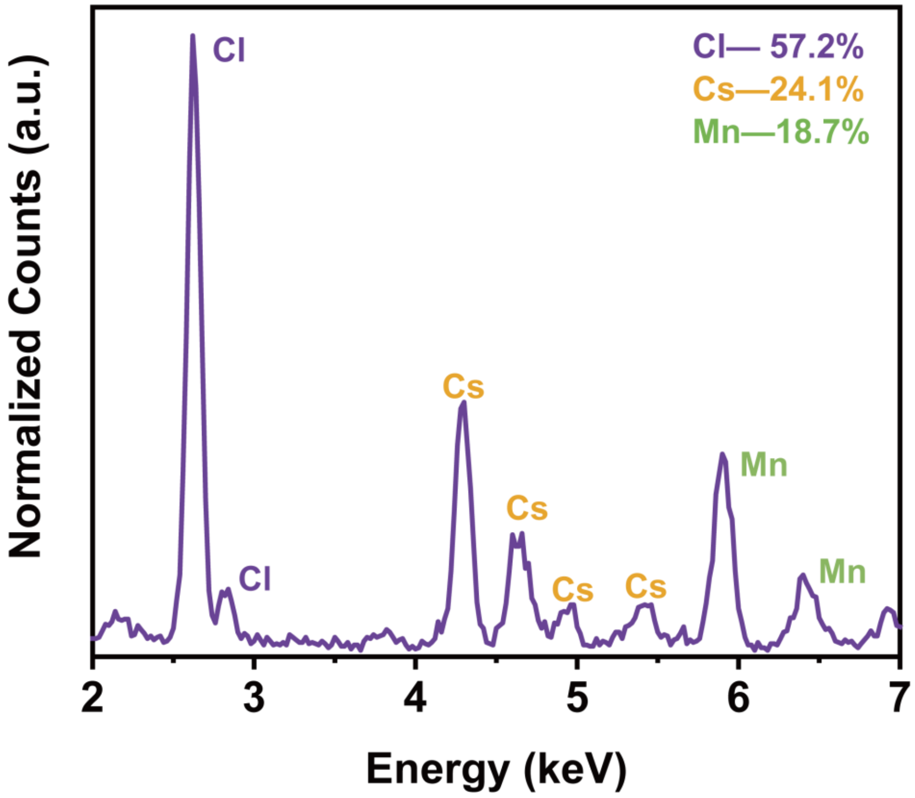

Figure S2. EDS spectra of $CsMnCl_3$ NCs. The element contents of Cs, Mn, and Cl are shown in the top right.

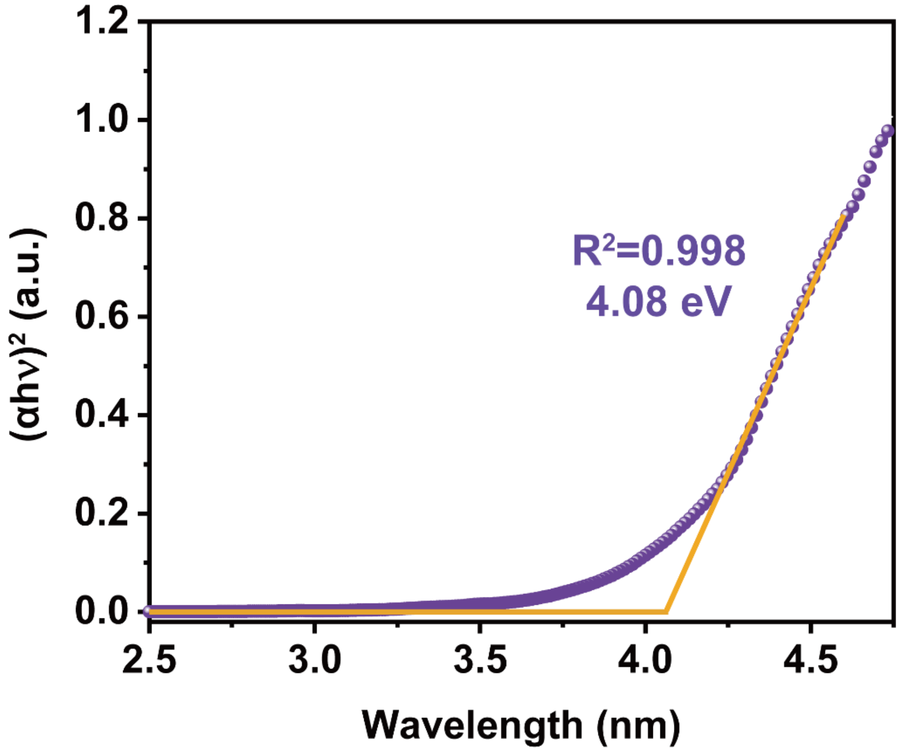

Figure S3. Tauc plot analysis of $CsMnCl_3$ NCs. A direct bandgap of 4.08 eV could be estimated.

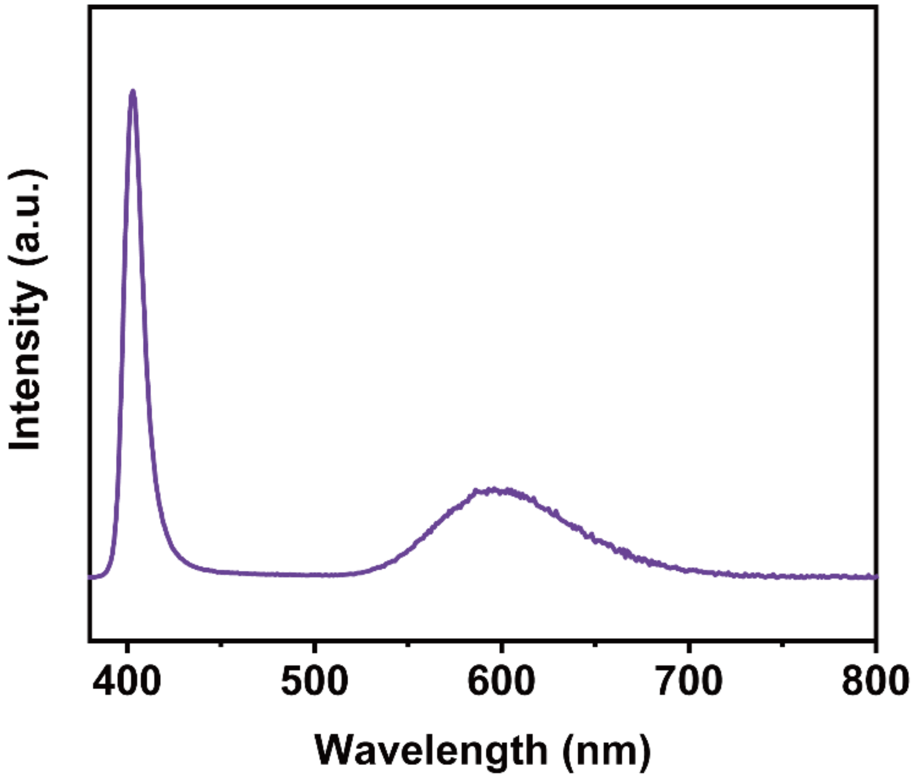

Figure S4. Photoluminescence spectra of $CsPbCl_3$:1.9%Mn NCs under 345 nm excitation. The emission from Mn^{2+} centered at 600 nm.

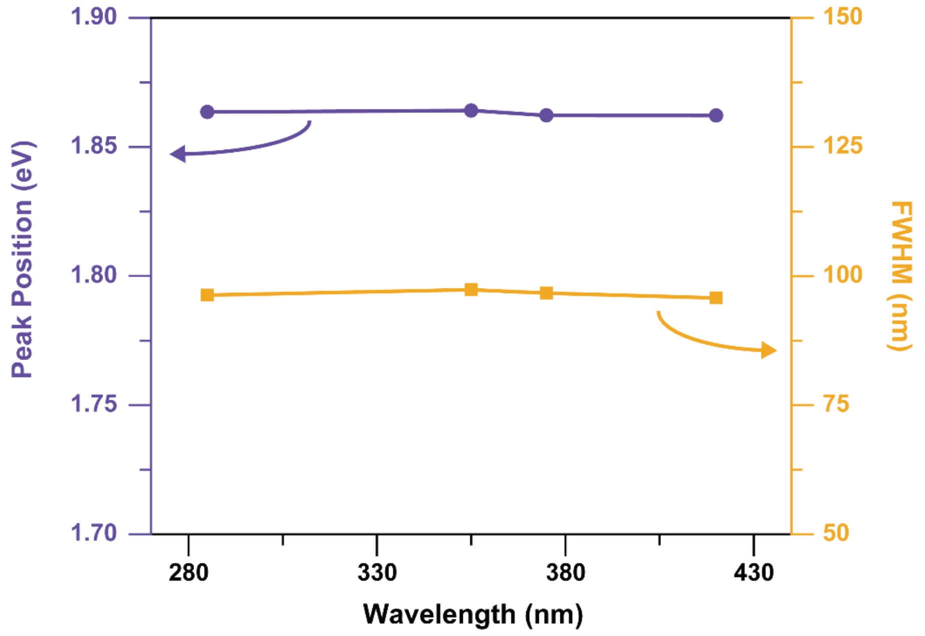

Figure S5. The PL peak position and FWHM *vs* excitation wavelength of $CsMnCl_3$ NCs.

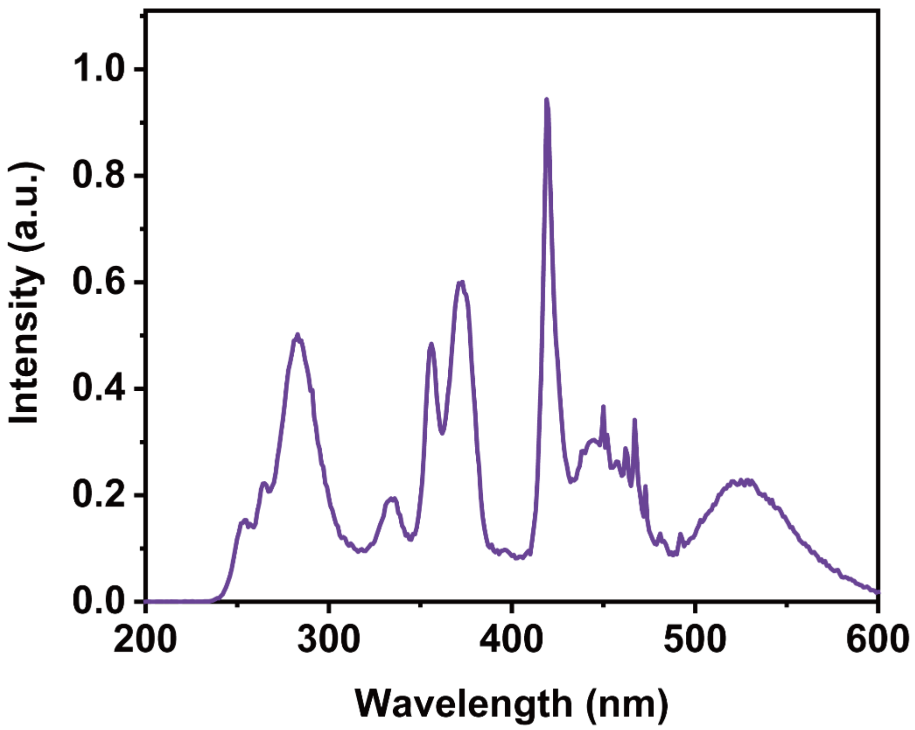

Figure S6. Excitation spectra of $CsMnCl_3$ NCs examined at 660 nm.

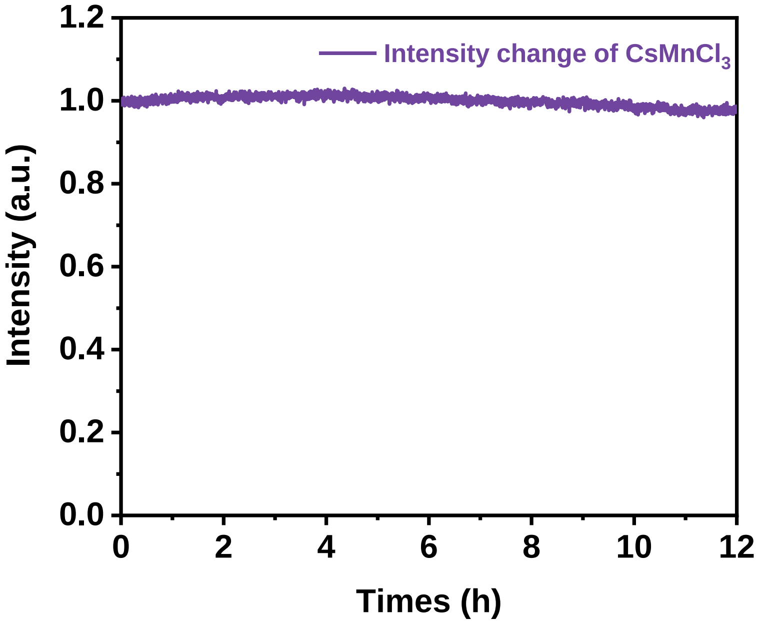

Figure S7. PL intensity at 660 nm for $CsMnCl_3$ NCs under excitation of 285 nm.

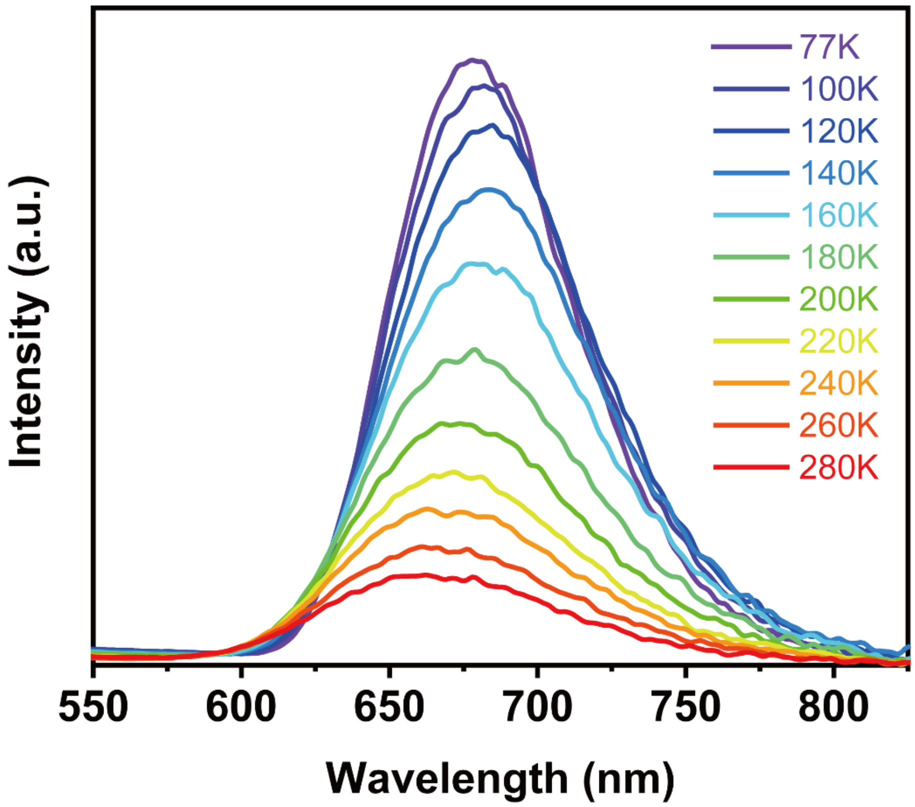

Figure S8. Temperature-dependent PL spectra of $CsMnCl_3$ NCs under excitation of 285 nm.

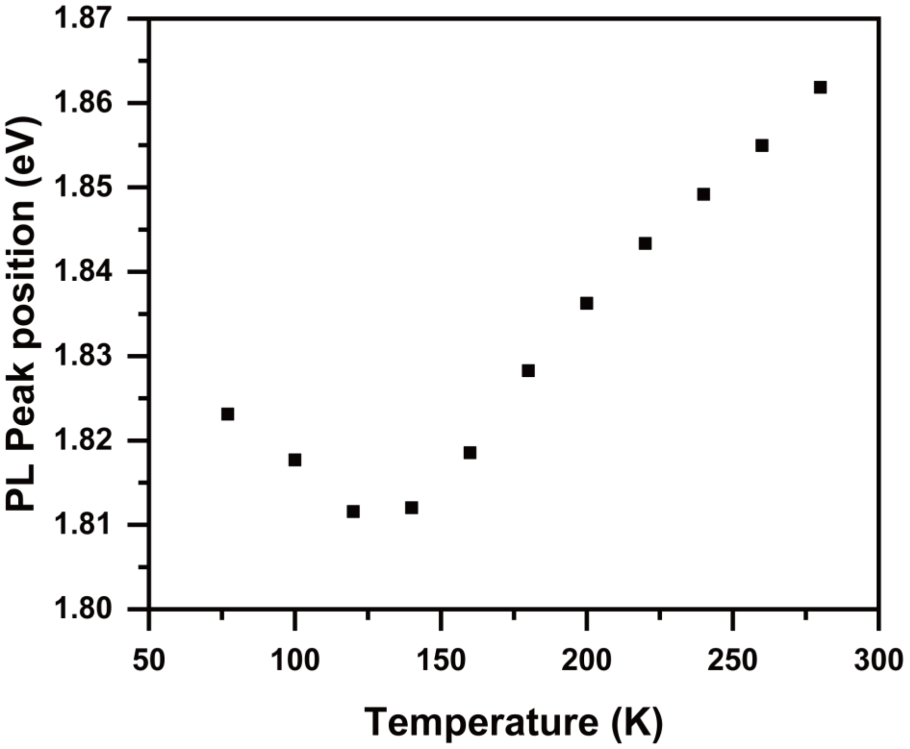

Figure S9. Temperature-dependent peak position of $CsMnCl_3$ NCs. The extreme value of peak position appears at around 120 K, which indicates a Mn-Mn magnetic coupling.

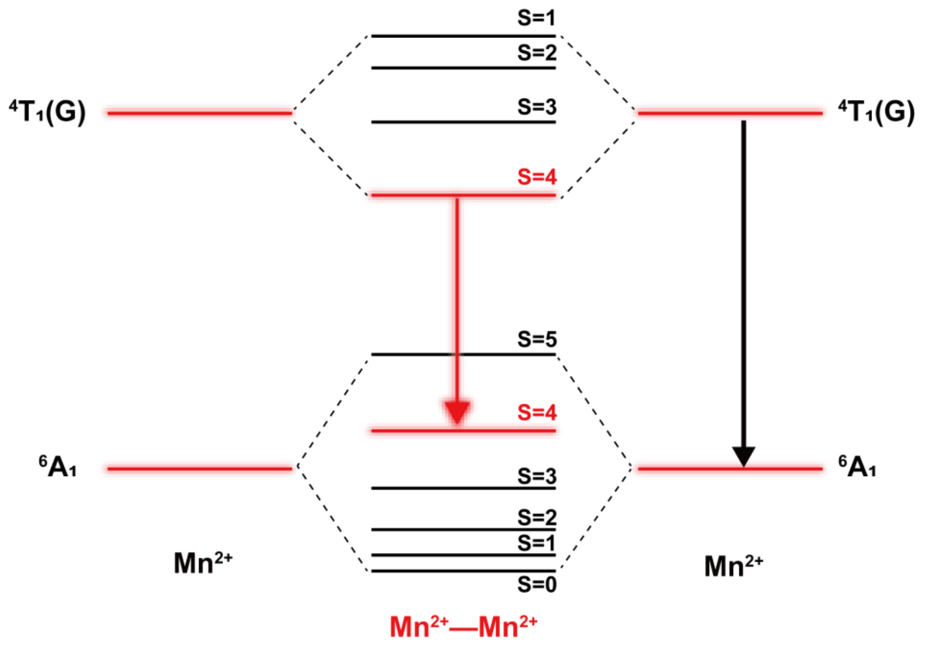

Figure S10. Schematic diagram of energy level splitting of Mn^{2+} in $CsMnCl_3$.

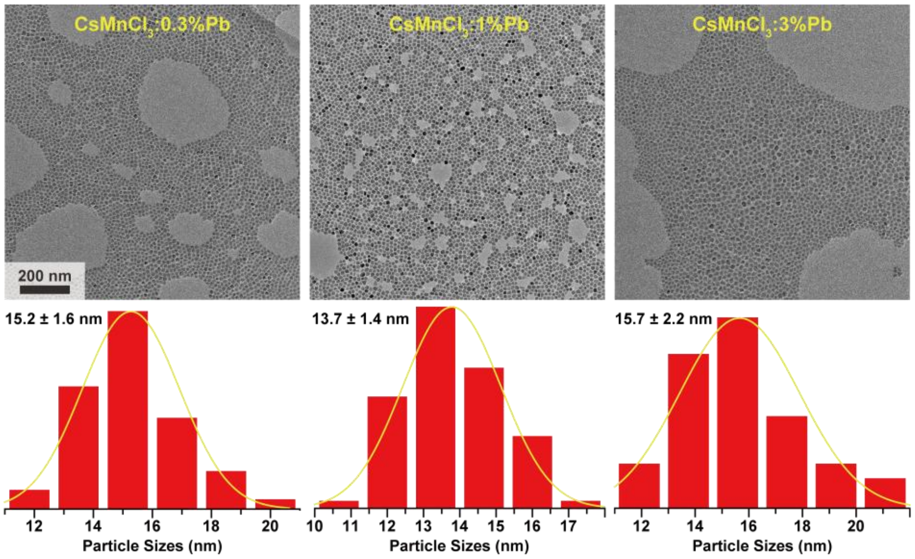

Figure S11. TEM images and corresponding size distributions of $CsMnCl_3$:Pb NCs.

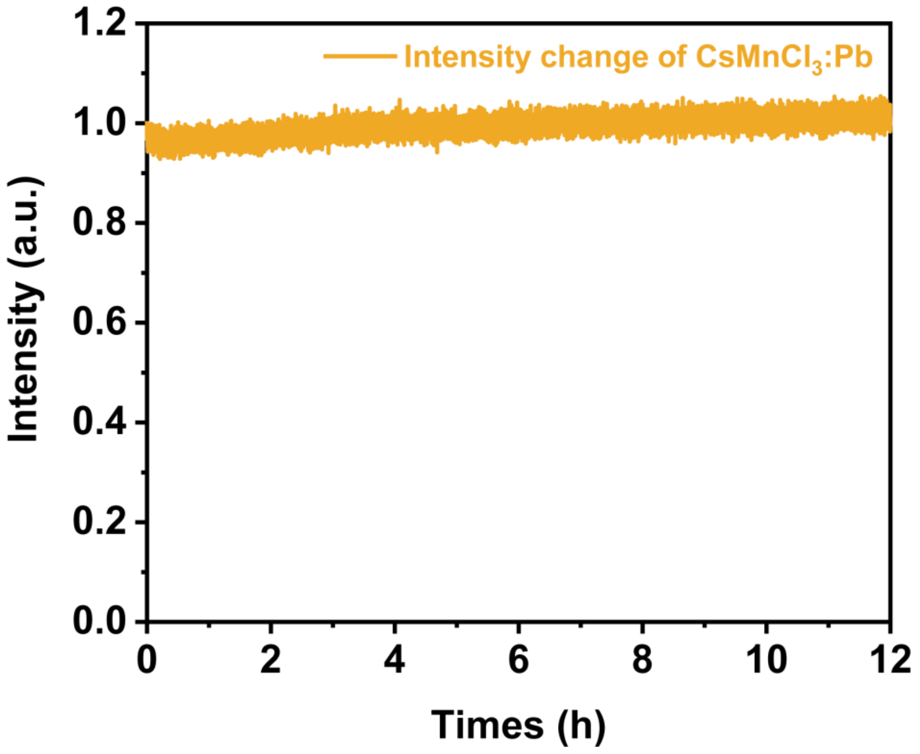

Figure S12. PL intensity at 660 nm for $CsMnCl_3$:1%Pb NCs under excitation of 285 nm.

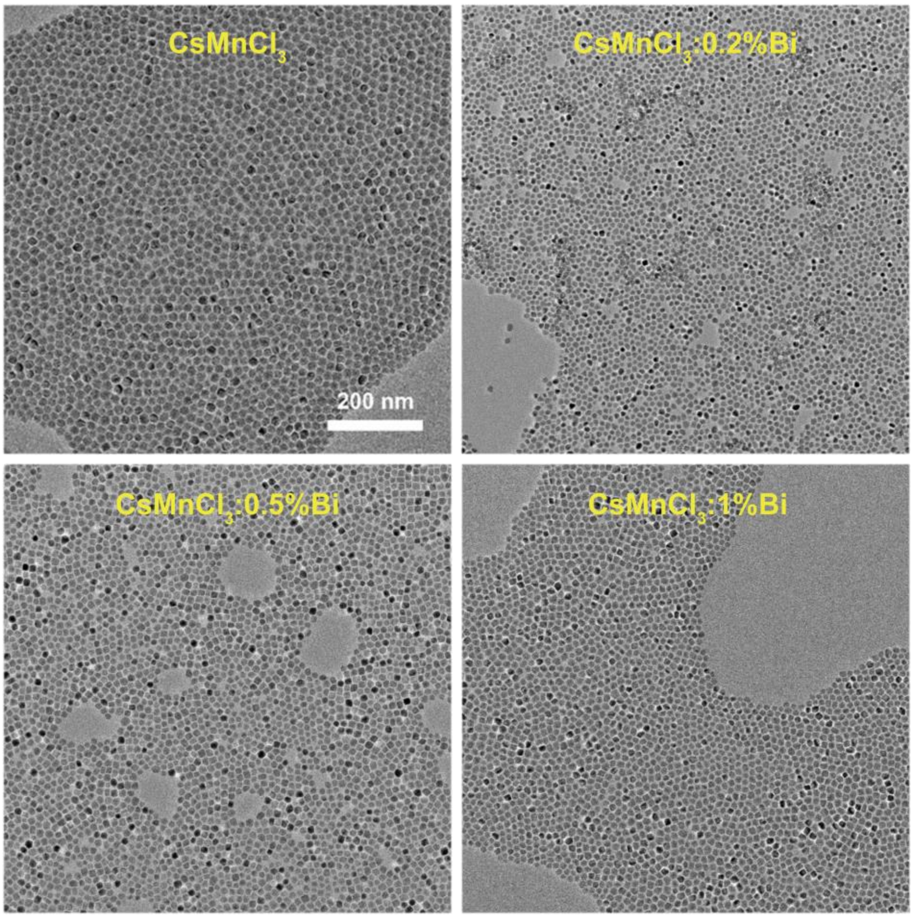

Figure S13. TEM images of $CsMnCl_3$:Bi NCs.

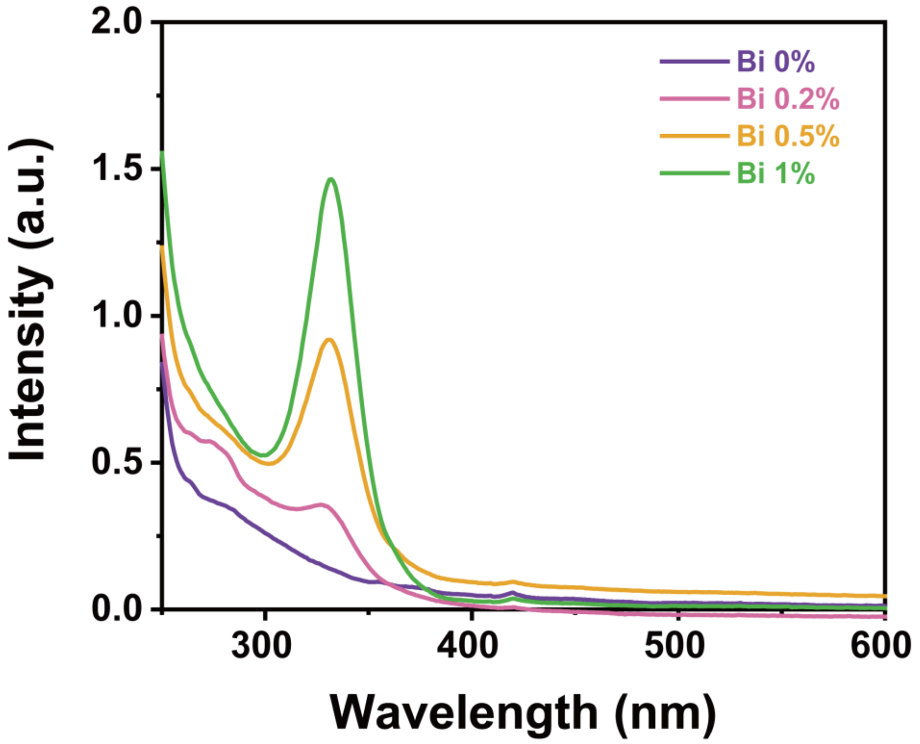

Figure S14. Absorption spectra of $CsMnCl_3$:Bi NCs.

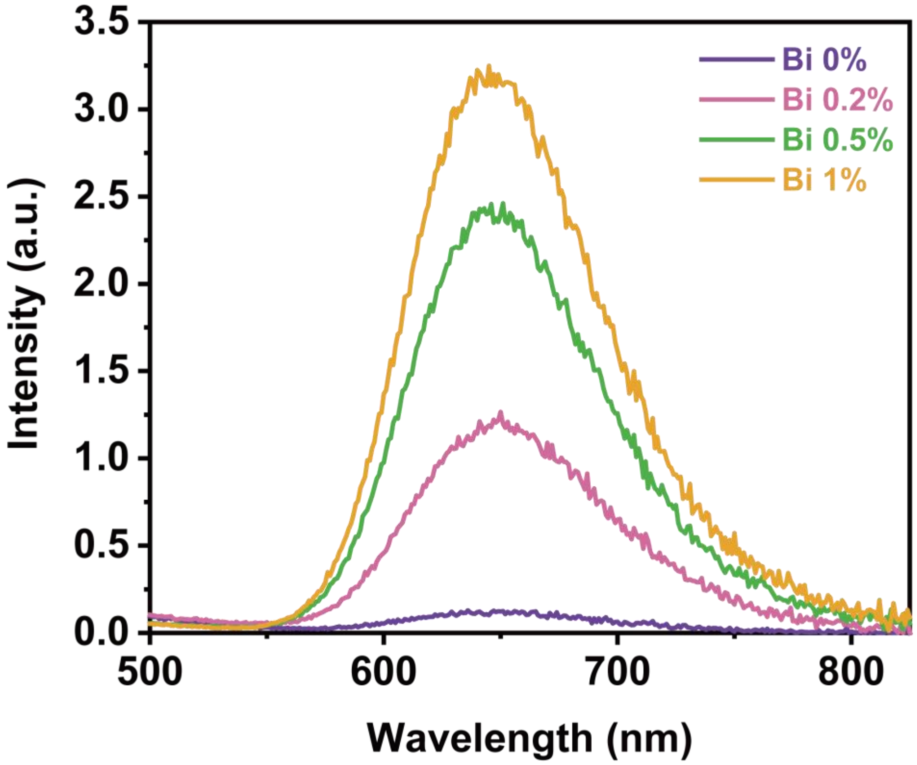

Figure S15. Photoluminscence spectra of $CsMnCl_3$:Bi NCs.

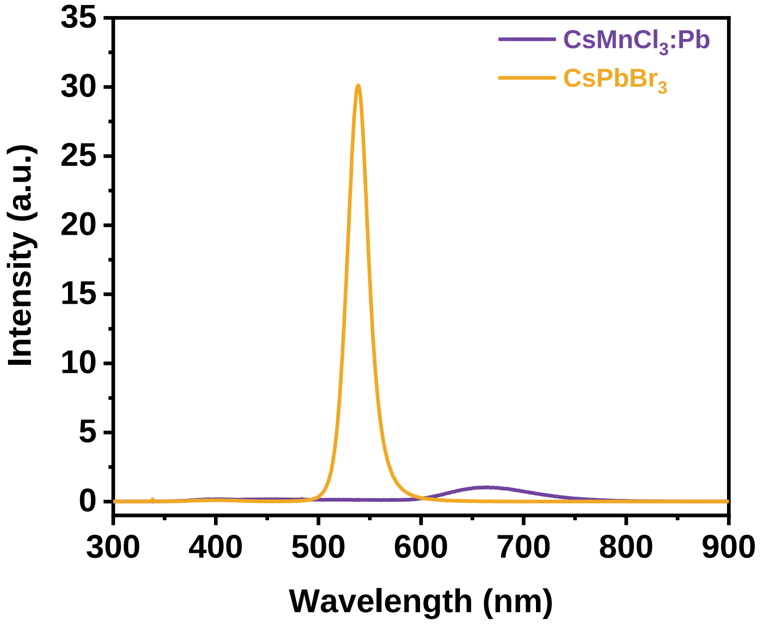

Figure S16. The radioluminescence spectra of $CsMnCl_3$:Pb and $CsPbBr_3$ NCs.

We use $CsPbBr_3$ QDs as a reference to estimate the light yield of $CsMnCl_3$:Pb NCs, which have a similar size and a known light yield about 21000 ph MeV^{-1}[3-5] according to the equation

$$\frac{LY_{CsMnCl_3:Pb}}{LY_{CsPbBr_3}} = \frac{R_{CsPbBr_3}}{R_{CsMnCl_3:Pb}} \times \frac{\int I_{CsMnCl_3:Pb}(\lambda)d\lambda \times S_{CsMnCl_3:Pb}}{\int I_{CsPbBr_3}(\lambda)d\lambda \times S_{CsPbBr_3}} \qquad (1)$$

where R is the X-ray deposited energy percentage of scintillators, I is the radioluminescence intensity at different wavelengths (λ), and S is the irradiation area. Due to the same sample thickness (about 800 μm, most of the X-ray photons were absorbed) and the same test condition. the light yield of $CsMnCl_3$:Pb NCs is estimate to 2500 ph MeV^{-1} according to the integrated intensity, which is 1/8 of $CsPbBr_3$ QDs.

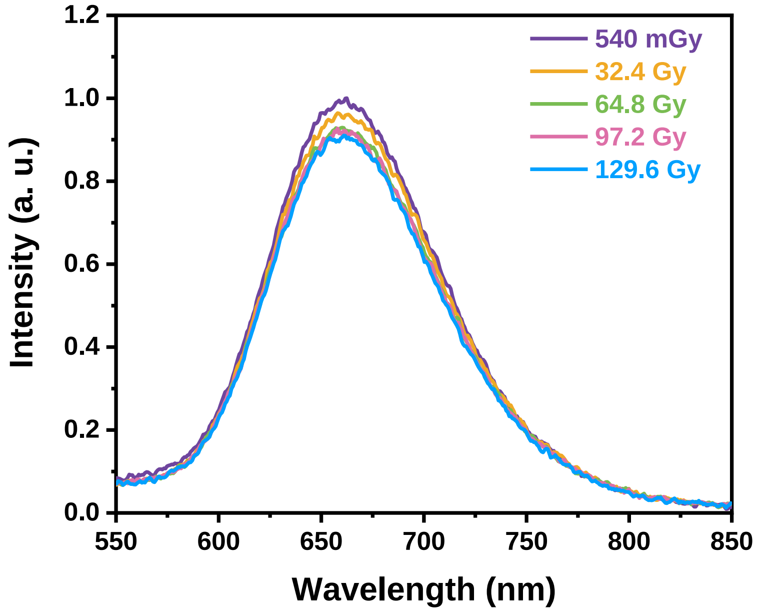

Figure S17. The radioluminescence spectra of $CsMnCl_3$:Pb NCs under irradiated with different X-ray total dose.

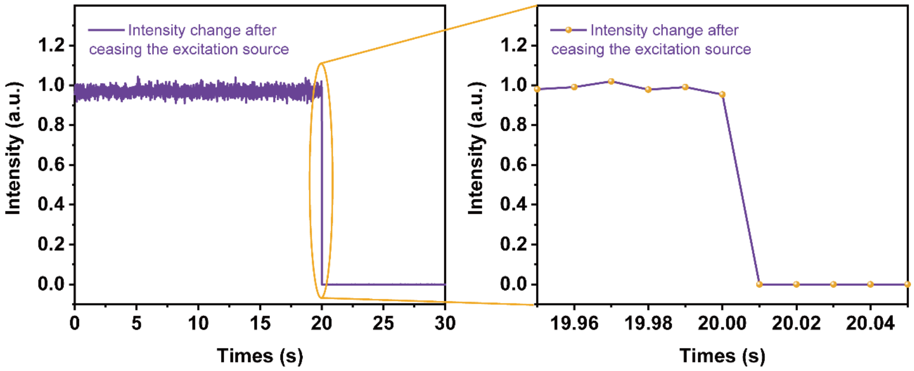

Figure S18. Afterglow intensities measurement of $CsMnCl_3$:Pb NCs after ceasing the excitation at 20 s and the details are shown in right.

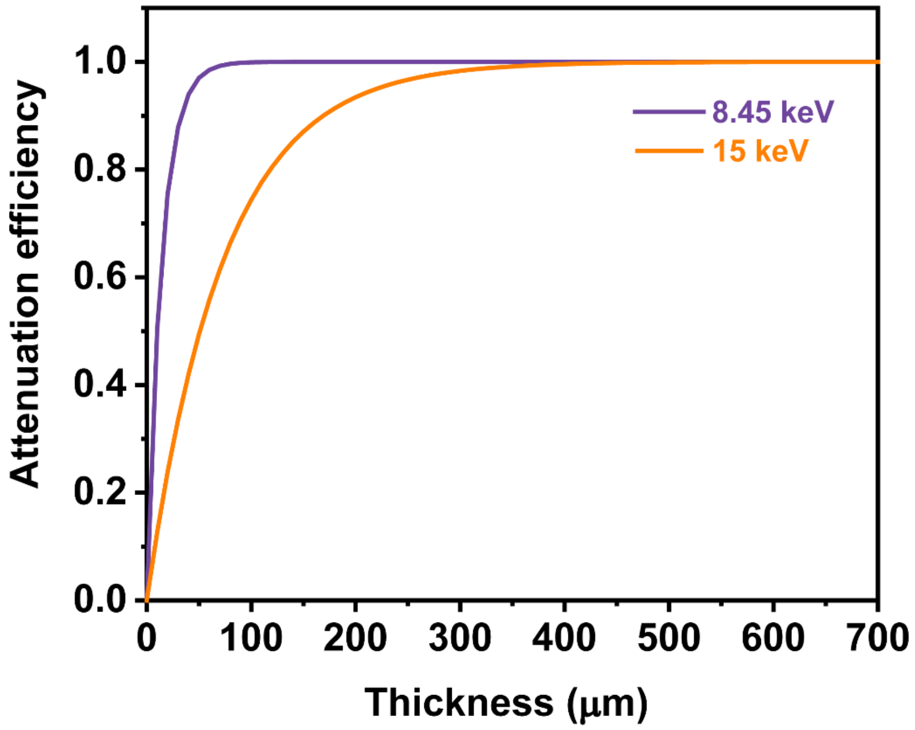

Figure S19. The relationship between attenuation efficiency and sample thickness. The X-ray energy are set as 8.45 keV (strongest) and 15 keV (average).

According to the Lambert–Beer law, we could estimate absorption efficiency as

$$I = I_O \times e^{-\mu x} \qquad (1)$$

where I and I_0 are the remainder and initial intensity, μ is the absorption coefficient and x is the material thickness. Almost all 8.45 keV X-rays and 96% 15 keV X-rays were absorbed with a 240 μm thickness.

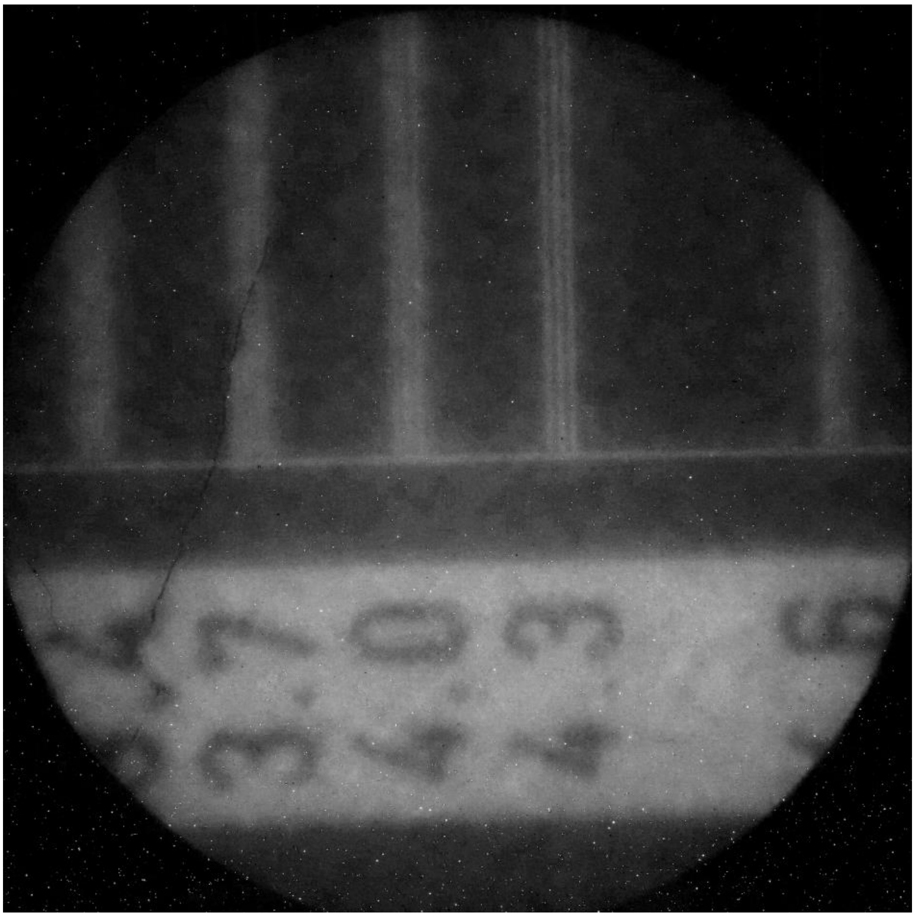

Figure S20. Spatial resolution measured by a line pair card.

Table S1. ICP-AES analysis of[Pb]/[Mn] ratio for $CsMnCl_3$:Pb NCs

Sample	Feed Ratio	Doping Ratio
$CsMnCl_3$	0	0
$CsMnCl_3$:0.3%Pb	0.3%	0.29%
$CsMnCl_3$:1%Pb	1%	1.2%
$CsMnCl_3$:3%Pb	3%	3.6%

Table S2. PLQY of $CsMnCl_3$:x% Pb nanocrystals, x=0, 0.3, 1, and 3.

Sample	PLQY
$CsMnCl_3$	0.7 ± 0.1%
$CsMnCl_3$:0.3%Pb	14.0 ± 0.2%
$CsMnCl_3$:1%Pb	21.1 ± 0.1%
$CsMnCl_3$:3%Pb	17.5 ± 0.5%

References

[1] L. Protesescu, S. Yakunin, M.I. Bodnarchuk, F. Krieg, R. Caputo, C. Hendon, R. Yang, A. Walsh, M.V. Kovalenko, *Nano Lett.* **2015**, *15*, 3692−3696.

[2] D. Parobek, B. Roman, Y. Dong, H. Jin, E. Lee, M. Sheldon, D. Son, *Nano Lett.* **2016**, *16*, 7376−7380.

[3] Y. Zhang, R. Sun, X. Ou, K. Fu, Q. Chen, Y. Ding, L.-J. Xu, L. Liu, Y. Han, A. V. Malko, X. Liu, H. Yang, O. M. Bakr, H. Liu, O. F. Mohammed, *ACS Nano* **2019**, *13*, 2520−2525.

[4] L.-J. Xu, X. Lin, Q. He, M. Worku, B. Ma, *Nat. Commun.* **2020**, 11, 4329−4355.

[5] B. Yang, L. Yin, G. Niu, J.-H. Yuan, K.-H. Xue, Z. Tan, X.-S. Miao, M. Niu, X. Du, H. Song, E. Lifshitz, J.Tang, *Adv. Mater.* **2019**, *31*, 1904711.
